# Sesamol Attenuates Neuroinflammation by Regulating the AMPK/SIRT1/NF-*κ*B Signaling Pathway after Spinal Cord Injury in Mice

**DOI:** 10.1155/2022/8010670

**Published:** 2022-01-06

**Authors:** Xiaochu Feng, Xianghang Chen, Muhammad Zaeem, Wanying Zhang, Liwan Song, Lulu Chen, Joana Mubwandarikwa, Xiangxiang Chen, Jian Xiao, Ling Xie, Keyong Ye

**Affiliations:** Department of Orthopaedics, Affiliated Pingyang Hospital and School of Pharmaceutical Science, Wenzhou Medical University, Wenzhou, Zhejiang 325000, China

## Abstract

Inflammation is one of the crucial mechanisms mediating spinal cord injury (SCI) progress. Sesamol, a component of sesame oil, has anti-inflammatory activity, but its mechanism in SCI remains unclear. We investigated if the AMPK/SIRT1/NF-*κ*B pathway participated in anti-inflammation of sesamol in SCI. Sesamol could inhibit neuronal apoptosis, reduce neuroinflammation, enhance M2 phenotype microglial polarization, and improved motor function recovery in mice after SCI. Furthermore, sesamol increased SIRT1 protein expression and p-AMPK/AMPK ratio, while it downregulated the p-p65/p65 ratio, indicating that sesamol treatment upregulated the AMPK/SIRT1 pathway and inhibited NF-*κ*B activation. However, these effects were blocked by compound C which is a specific AMPK inhibitor. Together, the study suggests that sesamol is a potential drug for antineuroinflammation and improving locomotor functional recovery through regulation of the AMPK/SIRT1/NF-*κ*B pathway in SCI.

## 1. Introduction

Spinal cord injury (SCI) is severe central nervous system (CNS) damage, and more than 250000 patients suffer from it every year [1]. However, there are few effective treatments for SCI. As a grievous neurological disease, SCI seriously destroys the function of motor and sensory neurons; paralysis caused by SCI brings huge medical and economic burden to the patients and their families [2, 3].

Traumatic SCI involves primary mechanical insult and the secondary injury [4]. The primary insult makes a direct crash to the spinal cord and damages cells, which also leads to a series of complex secondary injury molecular events, including toxic oxidative stress, excessive microglial activation, continuous inflammation, and rampant apoptosis [2, 5]. Neuroinflammation is one of the dominating mechanisms mediating secondary SCI progress. Microglia, the CNS-resident immune cells, maintain a resting state under control condition and become activated in response to local CNS injury [6]. There are two phenotypes of microglia called M1 and M2 after activation [7, 8]. M1 microglia are detrimental and can generate a lot of proinflammatory cytokines, including TNF-*α* and IL-6, while the M2 phenotype is protective and upregulates anti-inflammatory cytokines, including IL-10, which contribute to tissue regeneration and wound healing [9]. The resident microglia are activated after SCI, and then, a lot of inflammatory cytokines produced in the early stage of SCI, which will cause neuronal apoptosis, aggravate the injury and make recovery more difficult [10–12]. Therefore, it is especially important to alleviate inflammatory response in the early stage of injury.

Sesamol, a component of sesame oil [13], has been proven to have effects of anti-inflammation and neuroprotection [13–15]. As a member of the sirtuin (SIRTs) family, SIRT1 has been proven to have positive impacts on antiaging, anti-inflammation, and reducing oxidative stress damage [16–19]. AMP-activated protein kinase (AMPK) is a critical modulator of cellular energy, which coordinates numerous pathways to maintain energy homeostasis [20]. A number of evidences have indicated that AMPK upregulates protein expression of SIRT1 and inhibits inflammation under pathological conditions [16, 21, 22]. SIRT1, as a downstream protein of the AMPK signaling pathway, also plays a part in anti-inflammation via inhibiting NF-*κ*B [23–25]. The previous studies have proven that sesamol plays roles in anti-inflammation by suppressing NF-*κ*B activation and upregulating AMPK signaling [26, 27] and attenuating oxidative stress via activation of SIRT1 [28].

Sesamol has potential therapeutic use in SCI, but the underlying mechanism of sesamol in SCI remains poorly understood. This study confirmed the effect and mechanism of sesamol in anti-inflammation in mice following SCI and provided evidence for the potential clinical use of sesamol in SCI.

## 2. Materials and Methods

### 2.1. Animals

Healthy 8~10-week-old male C57BL/6J mice (weighing 20~25 g) were used in this study. All mice lived in standard housing conditions and can drink and eat freely. Animal experimental operations were performed according to protocols authorized by the Laboratory Animal Ethics Committee of Wenzhou Medical University (no. wydw2018-0043).

### 2.2. SCI and Drug Treatments

The SCI protocol for adult mice under sterile conditions is described previously. Shortly, animals were anesthetized with 1% pentobarbital sodium (50 mg/kg, i.p.) before surgery. Skin and muscles near spinous processes were incised to expose the dorsal cord in mice. After a laminectomy at the T9-T10 level, a 10 g weight from 20 mm height was fallen onto the exposed spinal cord by a modified New York University impactor to induce a moderate SCI [29]. As a control, mice in the sham group did not suffer from SCI after laminectomy and did not receive medication. After SCI surgery, the mouse bladders were manually emptied every morning and evening until bladder function returned to normal.

**Figure 1:**
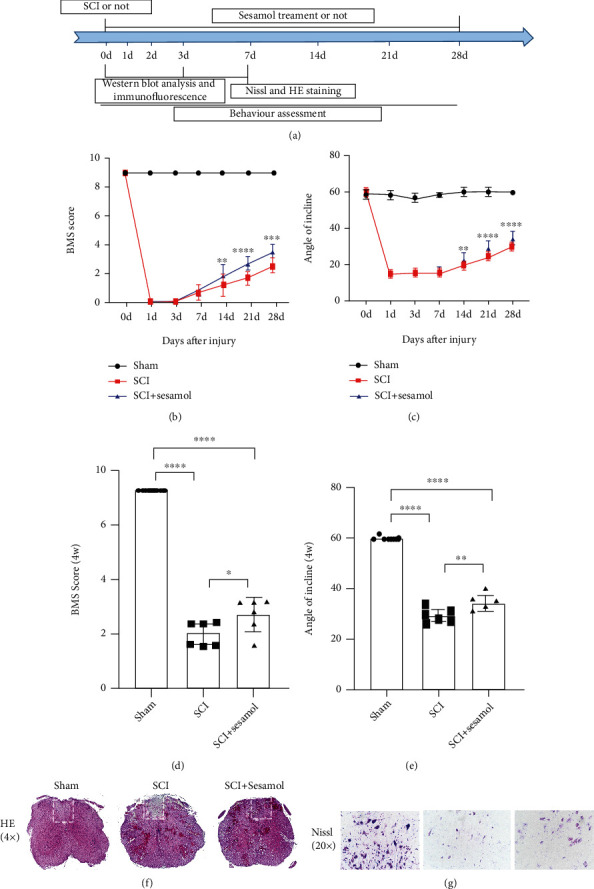
Sesamol evaluates histological outcomes and promotes locomotor functional restoration in mice suffering from SCI. (a) Time schedule of experimental design and analysis in mice. (b) The BMS scores of mice in each group at 0, 1, 3, 7, 14, 21, and 28 dpi. (c) Result of the inclined plane test of mice from different group at 28 dpi. (d, e) Quantitative analysis of the BMS score and incline angle at 28 dpi from (b) and (c), respectively (*n* = 6 per group). (f) H6istological assessment of the injured spinal cord by HE staining at 7 dpi. (g) Nissl staining of the injured spinal cord to evaluate the neuronal survival in each group at 7 dpi. Results are represented as mean ± SEM. ^#^*P* < 0.05, ^##^*P* < 0.01, ^###^*P* < 0.001, and ^####^*P* < 0.0001.

All mice are randomly assigned, and mice suffering from SCI were randomly arranged to two groups: SCI and SCI+sesamol. Mice in the SCI+sesamol group were administrated daily with sesamol (10 mg/kg, i.p.) dissolved in saline for 28 days, and animals in the SCI group received the same volume of normal saline every day. Mice were sacrificed, and the damaged spinal cord was taken out for analysis at the corresponding time point after SCI. The time points of administration and experimental arrangement for mice are shown in Figure 1(a).

### 2.3. Cell Culture and Intervention

BV2 cells were cultivated in DMEM supplemented with 10% fetal bovine serum and 1% penicillin and streptomycin mixture in a humidified incubator with 5% CO_2_ at 37°C. BV2 cells were arranged to control, lipopolysaccharide (LPS), LPS+sesamol, and compound C (LPS+sesamol+compound C) groups. Cells were incubated with 10 *μ*M sesamol dissolved in PBS for 2 h and then suffered from LPS (1 *μ*g/mL) for 24 h to stimulate inflammation. To explore whether the AMPK pathway participates in anti-inflammation of sesamol, cells were pretreated with compound C (a specific AMPK inhibitor) for 2 h before sesamol treatment.

### 2.4. Cell Viability Test

BV2 cells were incubated in a 96-well cell culture plate with 10000 cells per well for 24 h and then administrated with LPS combined with different concentrations of sesamol. The cell viability was detected with Cell Counting Kit-8 (CCK-8) according to the instructions. Shortly, after incubation, cells were further cultured for 2 h in 90 *μ*L of fresh DMEM supplemented with 10 *μ*L of CCK-8 solution. At last, optical density (OD) values at 450 nm were measured using a microplate reader. Six replicate wells of cells were arranged in each group.

### 2.5. Western Blot

After transcardial perfusion of saline, approximately 1 cm length of the spinal cord adjacent to the injury center was collected. Tissues and BV2 cells were lysed with a protein extraction reagent containing protease inhibitors and then centrifuged to get proteins. Whole tissue lysate (60 *μ*g) or cell lysate (20 *μ*g) was separated by 8%~12% SDS-PAGE, and then, proteins were blotted onto the PVDF membrane. Blocked with 5% (*w*/*v*) nonfat milk, the membrane was incubated at 4°C overnight using primary antibodies corresponding to these proteins: TNF-*α*, IL-6, Bax, CD86, CD206, p-AMPK (Thr172), AMPK, Bcl-2, SIRT1, p-p65 (S536), p65, and cleaved caspase-3, and further immersed in the corresponding HRP-conjugated secondary antibody for 1.5 h at room temperature (RT). At last, signal was visualized by using the ChemiDoc™ XRS^+^ imaging system (Bio-Rad), and the band intensity was analyzed by using Image Lab 5.2 software (Bio-Rad).

### 2.6. Immunofluorescence

For immunofluorescence, haematoxylin-eosin (HE), and Nissl staining, animals underwent transcardial perfusion with 4% (*w*/*v*) paraformaldehyde (PFA), and then, the injured spinal cord tissue was taken out and fixed with 4% PFA overnight. BV2 cell climbing slices were also fixed with 4% PFA for 15 min. The tissues were then dehydrated and immersed in paraffin. Next, tissues were sliced up to 5 *μ*m thickness of sections, and then, the slices were dewaxed using xylene and hydrated with 100, 90, 80, and 70% ethanol. After high-pressure antigen retrieval, tissue slices or BV2 cell climbing slices were blocked with 5% (*w*/*v*) BSA for 30 min at 37°C. Next, the slices were treated with primary antibodies at 4°C overnight as follows: NeuN, cleaved caspase-3, Iba1, CD206, SIRT1, and p65. Next, the sections or cell climbing slices were further incubated with a proper fluorescence-conjugated secondary antibody at RT for 1 h. Finally, DAPI was used for cellular nuclear staining. The images were visualized by using a Nikon ECLIPSE80i microscope.

### 2.7. HE Staining and Nissl Staining

Injured spinal cord sections were successively dewaxed and hydrated according to protocols described above. A part of tissue slices was used for HE staining with an HE kit, and other slices were stained with cresyl violet acetate for Nissl body staining according to the instructions. After staining, the slices were successively dehydrated and transparentized using 95% alcohol for 2 min and xylene for 5 min, respectively. Finally, the film was sealed with neutral resin. The damage area was photographed by an optical microscope (Olympus, Tokyo, Japan).

### 2.8. Locomotor Function Recovery Assessment

The hind limb locomotor function of mice was evaluated by the Basso Mouse Scale (BMS) scoring [30] and inclined plane test at 0, 1, 3, 7, 14, 21, and 28 days after injury (dpi). In short, animals were allowed to move freely a period of time in an empty room, and the BMS scores of mice were recorded according to the observation of paw posture, posterior ankle joint mobility, trunk stability, tail posture, and coordination. The BMS score of mice ranges from 0 (completely paralysis) to 9 (normal locomotion). For the inclined plane test, mice were placed on a rubber board, and then, the angles at which the mice could not hold its position for 5 sec were defined as the maximum angles, which could be used to evaluate the hind limb strength of mice [31]. The results of mouse hind limb movements were blindly recorded by two trained investigators.

### 2.9. ELISA

BV2 cells were coincubated with LPS combined with or without sesamol. IL-6 and TNF-*α* produced from cells were detected by using a commercial ELISA kit according to the instructions. The OD values at 450 nm and 630 nm were determined by using the SpectraMax microplate reader.

### 2.10. Statistical Analysis

Results are expressed as the mean ± SEM. The significant difference comparison of two groups was carried out by two-tailed Student's *t*-test. The statistical significance comparison of multiple groups was conducted by one-way analysis of variance (ANOVA) test with Tukey's multiple comparison test. All statistical analyses were conducted by using GraphPad Prism 8 software. *P* < 0.05 was regarded significant.

## 3. Results

### 3.1. Sesamol Improves Locomotor Functional Restoration in Mice after SCI

The BMS score and inclined plane test were applied to assess locomotor functional restoration, and HE staining as well as Nissl staining was carried out to evaluate histological outcomes in mice suffering from SCI. Prospectively, BMS scores of both injury groups were distinctly lower than that of the sham group, while injured mice treated with sesamol exhibited a distinct amelioration of posterior limb motor function with higher BMS scores and better coordinated crawling at 14, 21, and 28 dpi relative to mice suffering from SCI alone (Figures 1(b) and 1(c)). Cavity of necrotic tissue was detected by HE staining, and neuronal survival was measured using Nissl staining at 7 dpi, respectively. Result of HE staining displayed that sesamol treatment decreased SCI-induced cavity of necrotic tissue (Figure 1(f)). Similar to the result, sesamol reduced SCI-induced neuron loss; in other words, dramatically more survival neurons existed in the sesamol-treated group than in mice suffering from SCI alone detected by Nissl staining (Figure 1(g)). Collectively, the above data indicate that sesamol has a neuroprotective role in locomotor function recovery in mice after SCI.

### 3.2. Sesamol Attenuates Neuronal Apoptosis following SCI

**Figure 2:**
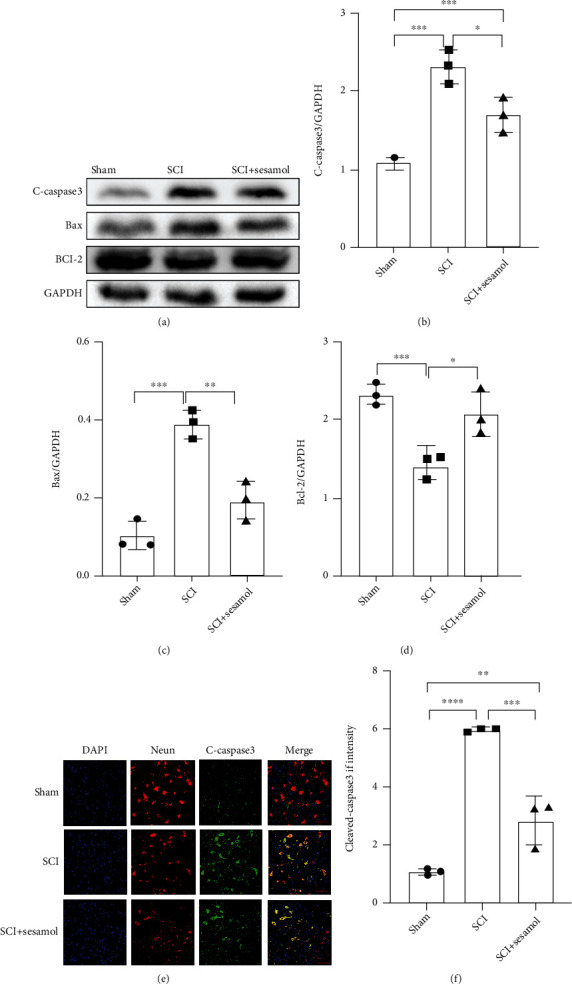
Sesamol protects neurons from apoptosis in mice after SCI. (a) Western blot images exhibiting apoptosis-related protein levels in the injured spinal cord of mice from each group at 7 dpi. (b–d) Quantitative analysis of the expression these proteins from (a) normalized to GAPDH. *n* = 3 per group. (e) Sesamol reduced the fluorescence degree of cleaved caspase-3 (green) in neurons (established by NeuN, red), and DAPI (blue) is for the nuclear staining (scale bar = 50 *μ*m). (f) Quantitative analysis of the fluorescence degree of cleaved caspase-3. Results are expressed as mean ± SEM (*n* = 3 per group). ^##^*P* < 0.05, ^###^*P* < 0.001, and ^####^*P* < 0.0001.

To confirm whether sesamol could reduce apoptosis induced by SCI, the level of apoptosis was measured by western blot and immunofluorescence at 7 dpi. Our results showed that traumatic SCI upregulated cleaved caspase-3 and Bax (markers of proapoptotic proteins) protein expression and weakened the protein level of Bcl-2 (an antiapoptotic protein), which were dramatically reversed by sesamol administration (Figures 2(a)–2(d)). These above data were confirmed by immunofluorescence staining of NeuN (marker for neuron) and cleaved caspase-3. Similarly, SCI increased cleaved caspase-3-positive neurons, but that was decreased by sesamol (Figure 2(e)). These results indicate that sesamol can prevent neuron apoptosis after SCI in vivo.

### 3.3. Sesamol Reduces Proinflammatory Cytokine Release and Regulates Phenotypic Polarization of Microglia

**Figure 3:**
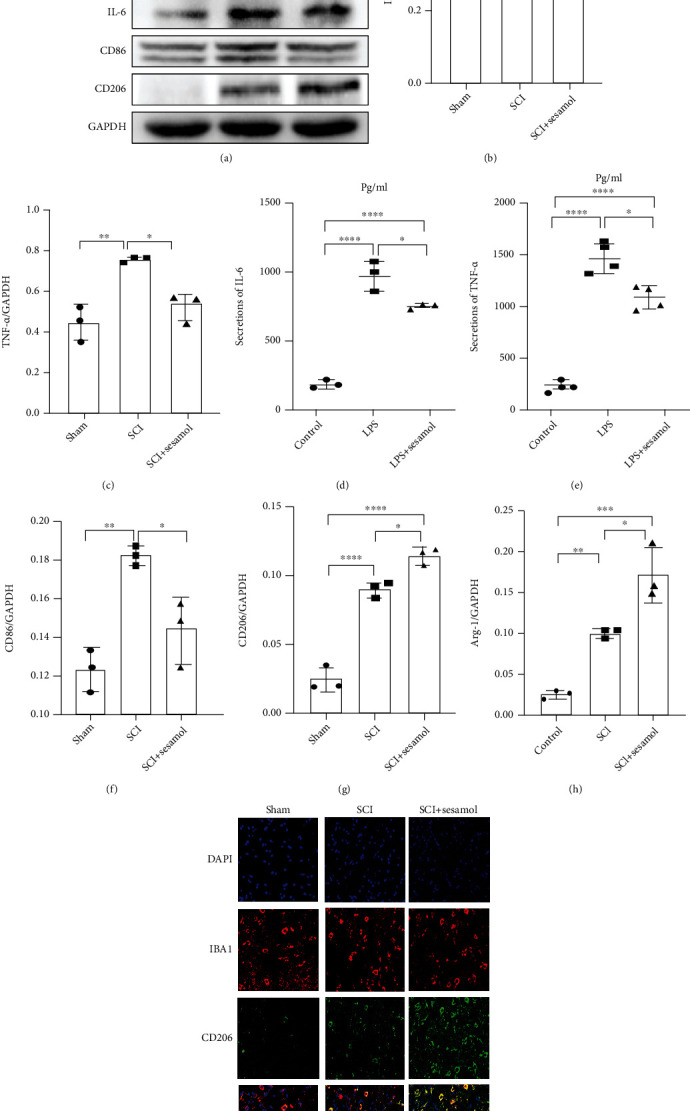
Sesamol attenuates inflammation and induces M2 phenotype microglial polarization in mice suffering from SCI. (a) Western blot images showing protein levels of IL-6, TNF-*α*, CD86, Arg-1, and CD206 in the injured spinal cord of mice at 3 dpi. (b, c) Quantification of TNF-*α* and IL-6 from (a), respectively (*n* = 3). (d, e) Statistical analysis of IL-6 and TNF-*α* concentrations determined by ELISA in BV2 cells suffering from LPS (*n* = 4 per group). (f–h) The quantitative analysis of CD86, CD206, and Arg-1 from (a) (*n* = 3 per group). (i) Sesamol increased the fluorescence degree of CD206 (green) in activated microglia (established by IBA1, red), and DAPI (blue) is for the nuclear staining (scale bar = 50 *μ*m). Values are represented as the mean ± SEM. ^#^*P* < 0.05, ^##^*P* < 0.01, ^###^*P* < 0.001, and ^####^*P* < 0.0001.

Accumulating evidences have revealed that inflammatory responses play key roles in secondary SCI. Therefore, levels of IL-6 and TNF-*α* were determined using western blot in vivo at 3 dpi and by ELISA in LPS-mediated BV2 cells. Compared with uninjured mice, acute SCI induced dramatic increase protein level of IL-6 and TNF-*α* in the damaged spinal cord, while the level of cytokines was significantly decreased by sesamol (Figures 3(a)–3(c)). Consistent with the result, increase in TNF-*α* and IL-6 was also distinctly overturned by sesamol in LPS-induced BV2 cells (Figures 3(d) and 3(e)).

To further understand the anti-inflammation role of sesamol in mice after SCI, phenotypic polarization of microglia was determined at 3 dpi. The data obtained by western blot analysis showed that sesamol administration prevented M1 microglia-related protein (CD86) expression and increased protein levels of M2 microglial mediators, including CD206 and arginase 1 (Arg1) (Figures 3(a) and 3(f)–3(h)). Consistent with the results, immunofluorescence analysis also verified that sesamol improved the CD206 protein expression in IBA1^+^ microglia after SCI (Figure 3(i)). These above data indicate that sesamol inhibits production of proinflammatory factors and induces M2 phenotype microglial polarization.

### 3.4. Effect of Sesamol on AMPK/SIRT1/NF-*κ*B Pathways in SCI

**Figure 4:**
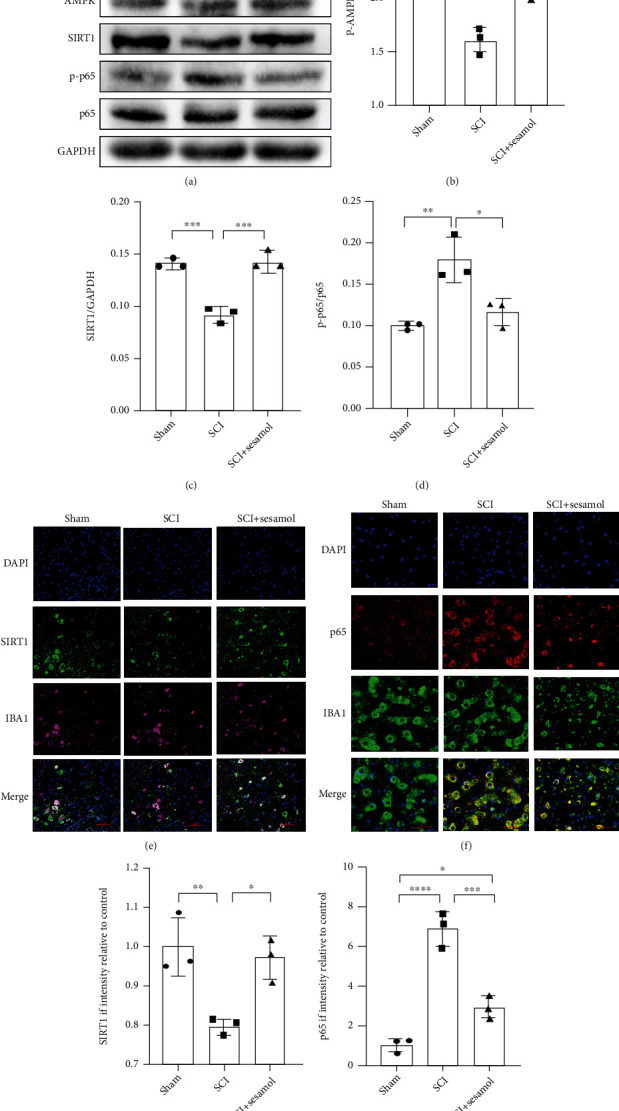
Sesamol regulates AMPK/SIRT1/NF-*κ*B pathways in mice following SCI. (a) Western blot images showing protein expressions of p-AMPK, AMPK, SIRT1, p-p65, and p65 in the spinal cord of mice from uninjured, SCI, and sesamol-treated group at 3 dpi. (b–d) Quantitative analysis of western blot results from (a) (*n* = 3 per group). (e) Representative images showing that sesamol increased the fluorescence degree of SIRT1 (green) in activated microglia (established by IBA1, purple), and DAPI (blue) is for the nuclear staining (scale bar = 50 *μ*m). (f) Sesamol reduced the fluorescence degree of p65 (red) in activated microglia (scale bar = 50 *μ*m). (g, h) Quantification of the fluorescence degree of SIRT1 and p65 in activated microglia from (e) and (f), respectively. Data are represented as the mean ± SEM (*n* = 3 per group). ^##^*P* < 0.01, ^###^*P* < 0.001, and ^####^*P* < 0.0001.

To understand the underlying mechanism of sesamol alleviating inflammation and promoting SCI restoration, effects of sesamol on regulating AMPK/SIRT1/NF-*κ*B pathways at 3 dpi were evaluated. Compared with uninjured mice, SCI resulted in significant decreases in SIRT1 protein expression and the p-AMPK/AMPK ratio, which was reversed by sesamol treatment (Figures 4(a)–4(c)). Activation of NF-*κ*B is connected with proinflammatory factor secretion and phenotypic changes of microglia [32]; thus, protein level of NF-*κ*B p65 and its phosphorylation level (p-p65) were determined by western blot analysis. The result exhibited that the ratio of p-p65/p65 was remarkably increased in mice following SCI relative to uninjured mice, which was dramatically inhibited by sesamol administration (Figures 4(a) and 4(d)). Similar to the result, SIRT1 was upregulated and P65 was downregulated in Iba1^+^ microglia after SCI with sesamol administration (Figures 4(e) and 4(f)). These above data indicate that sesamol may activate AMPK/SIRT1 and suppress activation of NF-*κ*B pathways in mice after SCI.

### 3.5. AMPK Participates in Sesamol Alleviating Inflammation in LPS-Stimulated Microglia

**Figure 5:**
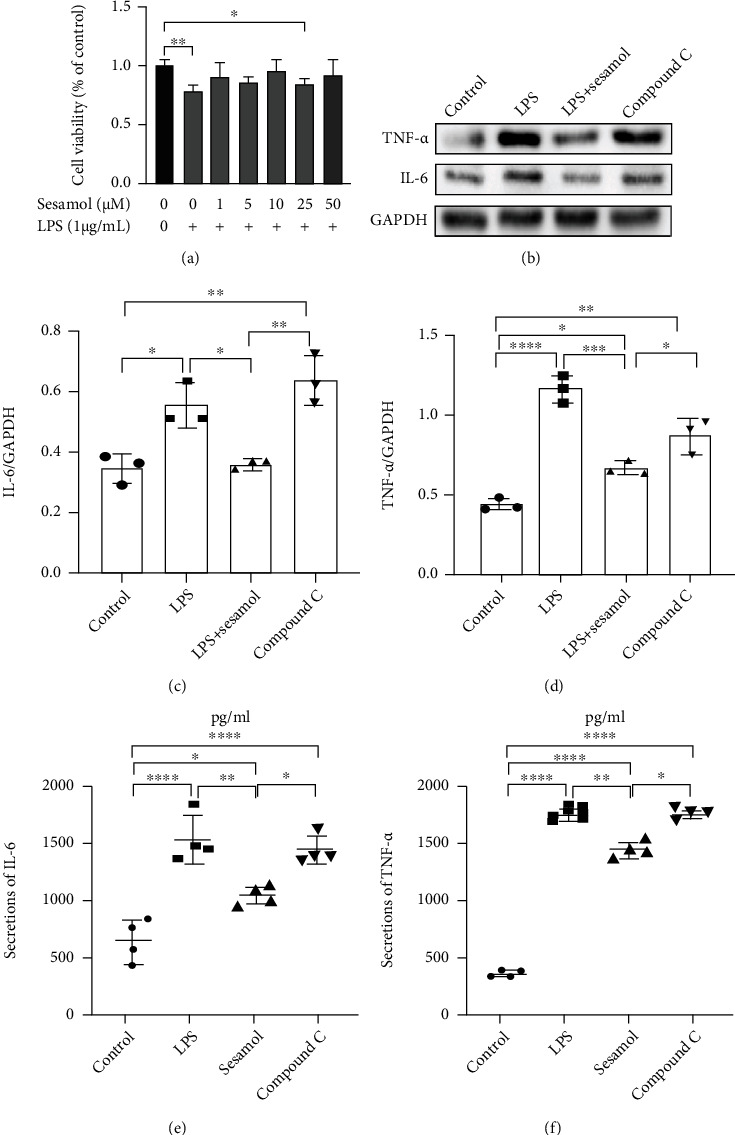
Sesamol-reduced proinflammatory cytokine secretions are reversed by compound C in LPS-mediated BV2 cells. (a) Quantification cell viability of BV2 cells incubated with LPS or (and) sesamol determined by using the CCK-8 kit. (b) Western blot images showing protein expressions of TNF-*α* and IL-6 in BV2 cells exposed to different conditions. (c, d) Quantitative assessment of TNF-*α* and IL-6 from (a), respectively (*n* = 3 per group). (e, f) Statistical analysis of IL-6 and TNF-*α* concentrations determined using ELISA in BV2 cells suffering from different conditions (*n* = 4 per group). Values are expressed as mean ± SEM. ^#^*P* < 0.05, ^##^*P* < 0.01, ^###^*P* < 0.001, and ^####^*P* < 0.0001.

To further confirm whether sesamol inhibited inflammation by activating the AMPK pathway following SCI, a specific AMPK inhibitor (compound C) was applied in LPS-induced BV2 cells. Then, protein expression of proinflammatory cytokines was examined by western blot and ELISA. Results in Figures 5(a)–5(e) showed that sesamol (10 *μ*M) significantly reduced the protein expression of IL-6 and TNF-*α* in LPS-induced BV2 cells, which was reversed by compound C (5 *μ*M). Collectively, these results reveal that AMPK may participate in anti-inflammation of sesamol in microglia stimulated by LPS.

### 3.6. AMPK Activation Participates in Sesamol-Induced M2 Microglial Polarization

**Figure 6:**
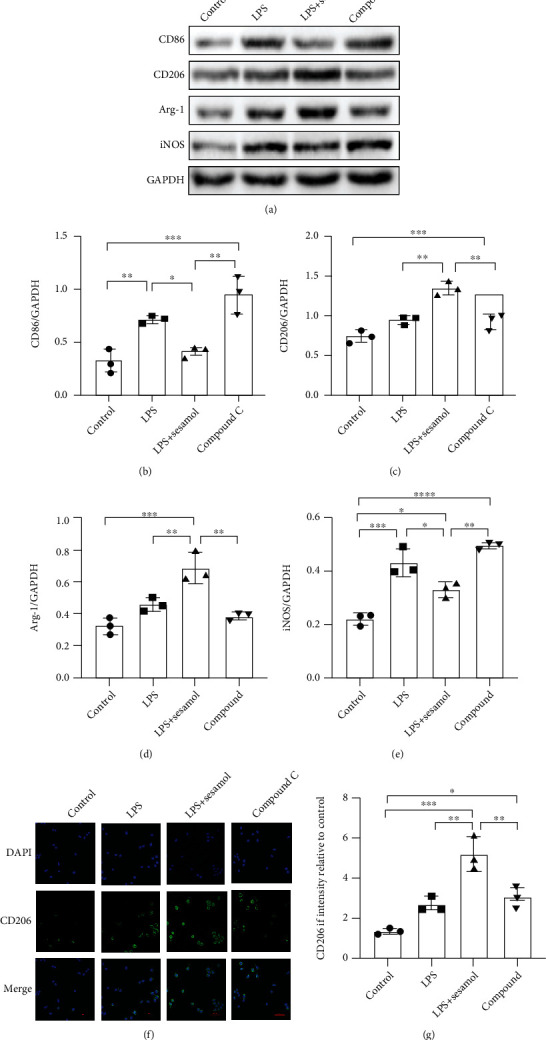
AMPK participates in sesamol-stimulated M2 microglial polarization. (a) Western blot images showing that compound C reversed effects of sesamol on protein levels of CD86, iNOS, Arg-1, and CD206 in BV2 cells. (b–e) Quantitative analysis of CD86, CD206, Arg-1, and iNOS from (a) normalized to GAPDH. (f) Compound C overturned fluorescence intensity of CD206 (green) increased by sesamol in microglia (scale bar = 50 *μ*m). (g) Quantification of the fluorescence degree of CD206 from (f). Results are represented as the mean ± SEM (*n* = 3 per group). ^#^*P* < 0.05, ^##^*P* < 0.01, ^###^*P* < 0.001, and ^####^*P* < 0.0001.

M1 phenotype microglia have been proven to be neurotoxic and induce inflammation response, while M2 microglia perform anti-inflammation. In this study, western blot and immunofluorescence were applied to further determine whether AMPK activation participates in sesamol-induced M2 microglial polarization in vitro. The results showed that both CD86 (a M1 microglia indicator) and CD206 (an established marker for M2 microglia) were significantly increased in LPS-stimulated BV2 cells. However, sesamol administration decreased dramatically CD86 protein level and further induced CD206 protein expression, which were significantly reversed by the AMPK inhibitor compound C (Figures 6(a)–6(e)). Consistent with the result, fluorescence intensity of CD206 in LPS-mediated BV2 cells was also remarkably increased by sesamol treatment, which was notably reduced by compound C (Figure 6(f)). These results confirm that sesamol promotes M2 polarization in BV2 cells partly regulated by the AMPK pathway.

### 3.7. Sesamol Alleviating Inflammation Response in Microglia Is Regulated via the AMPK/SIRT1/NF-*κ*B p65 Pathway

**Figure 7:**
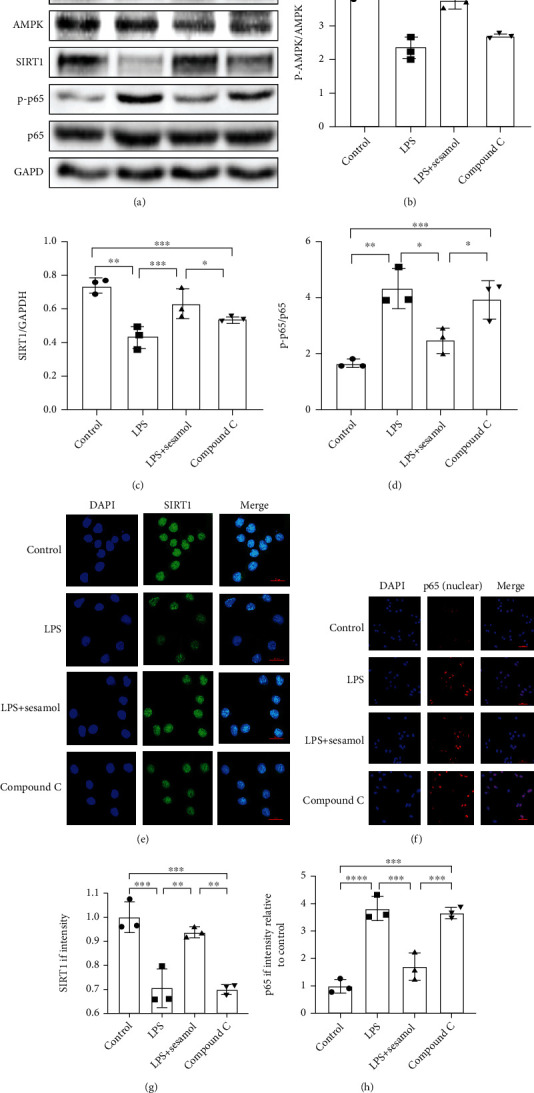
Sesamol regulates the AMPK/SIRT1/NF-*κ*B pathway in BV2 cells suffering from LPS. (a) Western blot images showing that compound C reversed p-AMPK, AMPK, SIRT1, p-p65, and p65 protein expression regulated by sesamol in BV2 cells incubated with LPS. (b–d) Quantification of p-AMPK/AMPK ratio, SIRT1, and p-p65/p65 ratio data from (a) (*n* = 3 per group). (e) Representative images showing that compound C counteracted fluorescence intensity of SIRT1 (green) increased by sesamol (scale bar = 20 *μ*m). (f) Representative images showing that sesamol decreased fluorescence intensity of nuclear p65 (red) while it was reversed by compound C (scale bar = 50 *μ*m). (g, h) Quantification of the fluorescence degree of SIRT1 and p65 in BV2 from (e) and (f), respectively (*n* = 3 per group). Results are represented as the mean ± SEM. ^#^*P* < 0.05, ^##^*P* < 0.01, ^###^*P* < 0.001, and ^####^*P* < 0.0001.

Evidences have shown that SIRT1 is a downstream target of the AMPK signaling pathway and participates in anti-inflammation via suppression of NF-*κ*B p65 activation [23–25]. To further verify sesamol playing an anti-inflammatory role in microglia via the AMPK/SIRT1/NF-*κ*B pathway, compound C was applied in BV2 cells, and then, protein expression of SIRT1, AMPK, and NF-*κ*B was analyzed using western blot. Results from Figures 7(a)–7(d) showed that the SIRT1 protein level and p-AMPK/AMPK ratio increased significantly while the p-p65/p65 ratio reduced remarkably in sesamol-treated BV2 cells compared with cells exposed to LPS only. However, these trends caused by sesamol were apparently overturned by compound C. Moreover, sesamol also resulted in distinct enhancement of fluorescence intensity of SIRT1 and a remarkable reduction of p65 fluorescence intensity, which were reversed by compound C (Figures 7(e) and 7(f)). Combined with data from Figure 5, these data further confirm that sesamol prevents inflammation through regulation of the AMPK/SIRT1/NF-*κ*B pathway.

## 4. Discussion

Secondary SCI is accompanied by long-term neuroinflammation, oxidative stress, and apoptosis [33]. Sesamol is a kind of excipient used in food and medicine and plays roles in antioxidant stress, antiaging, and anti-inflammation [34–36]. Nevertheless, the protective roles and the underlying mechanism of sesamol in SCI repair remain poorly understood. The current study examined the neuroprotective, antineuroinflammatory, and antiapoptotic roles of sesamol in mice following SCI and further explored the underlying mechanisms both in mice and in BV2 cells. Our results indicate that sesamol can ameliorate neuronal apoptosis, reduce inflammatory response, promote M2 microglial polarization, and improve neurological function restoration in mice after SCI. Furthermore, our data provide mechanistic evidence for the hypothesis that the AMPK/SIRT1/NF-*κ*B pathway might be important to the antineuroinflammatory ability of sesamol in microglia.

SCI leads to direct or indirect damage to neurons, causing rampant neuronal apoptosis at early stages of injury, which is one of key obstacles to the SCI restoration. Thus, preventing neuron against apoptosis is believed to be an effective approach to promote neural restoration after SCI. In the current study, sesamol could promote locomotor functional recovery and histological outcomes in mice after SCI. Our further results demonstrated that sesamol caused dramatic reduction of cleaved caspase-3 and Bax but a significant upregulation of Bcl-2, particularly within neurons in mice after SCI. Ample evidences indicate that Bax and cleaved caspase-3 participate in proapoptosis, while Bcl-2 has an antiapoptotic role [29, 37]. This work provides novel evidence that sesamol plays an important role of antiapoptosis within neurons in mice suffering from SCI.

Immune response is activated after SCI, and NF-*κ*B (p65) is one of the important regulators participating in infiltration of monocytes, neutrophils, and activated microglia into the damaged site and secretion of a lot of proinflammatory cytokines, including IL-6 and TNF-*α* [38, 39]. Ample evidences have demonstrated that microglia in CNS contribute to neuroinflammation progress [40, 41]. Our results revealed that sesamol significantly reduced TNF-*α* and IL-6 protein levels in mice suffering from SCI and in LPS-mediated microglia. This is consistent with the previous study showing that sesamol inhibits dextran sulphate sodium-induced inflammation in colitis mice [35]. After CNS injury, microglia are activated and polarized to the M1 phenotype with proinflammatory function and the M2 phenotype with anti-inflammatory effect [42, 43]. However, whether sesamol alleviates neuroinflammation related to regulating polarization of the microglial phenotype is still unknown. In our study, the protein level of CD86, an indicator of M1 microglia, was significantly increased after SCI and then dramatically reduced after sesamol administration. In contrast, the protein levels of Arg-1 and CD206 specially expressed in M2 microglia were increased at early days after SCI and further increased after sesamol administration. These data indicate that sesamol treatment decreases the proportion of M1 but increases M2 phenotype cells in activated microglia, which may contribute to antineuroinflammation.

Sesamol is extracted from sesame oil and can be used as an antioxidant in foods and medicines [44]. Several studies have revealed that sesamol protects the body from various disease conditions such as obesity, hyperlipidemia, and diabetic foot ulcer mainly through regulating lipid as well as energy metabolism and reducing inflammatory cell infiltration [45–47]. Ample previous studies have also documented that sesamol has anti-inflammatory effects via upregulating AMPK signaling and inhibiting NF-*κ*B activation [26, 27]. Moreover, sesamol can activate SIRT1 signaling to attenuate oxidative stress which will lead to cell apoptosis [28]. It is well known that AMPK is a key energy sensor that mediates cellular energy balance in the body [48]. AMPK also is found to be an important regulator participating in the macrophage phenotype [49]. SIRT1, a member of the SIRT family, has antiaging and anti-inflammatory effects [50]. SIRT1 acts as a downstream target of the AMPK pathway, and it also inhibits the activation of NF-*κ*B [27]. Many evidences verify the inhibitory effect of the AMPK/SIRT1/NF-*κ*B pathway on inflammation [22, 25, 48]. In the present study, compound C, a specific inhibitor of AMPK, was used in BV2 cells suffering from LPS. Our results reveal that AMPK participates in inhibitory effect of sesamol on inflammation. Moreover, our data found that sesamol reduced protein levels of CD86 and iNOS which established M1 microglia, while it increased M2 phenotype microglial protein (CD206 and Arg-1) expressions in BV2 cells exposed to LPS, but these results could be reversed by compound C. Microglia are activated as M1-type microglia after SCI and then secrete proinflammatory factors, which aggravate neuronal apoptosis in an inflammatory environment and make tissue repair difficult [51]. Excitingly, sesamol can promote M2 phenotype polarization and decrease proinflammatory cytokine secretion, sequentially improve the inflammatory environment around the tissue, and consequently inhibit neuronal apoptosis and exert a neuroprotective role in mice following SCI. Finally, this study confirmed that sesamol could activate the AMPK/SIRT1 pathway and inhibit NF-*κ*B activation in vitro (Figure 7). Therefore, this study indicates that sesamol attenuates neuroinflammation and improves neuron survival partly through regulating the AMPK/SIRT1/NF-*κ*B pathway in mice following SCI.

## 5. Conclusion

This study suggests that sesamol promotes polarization of M2 phenotype microglia, inhibits neuroinflammation, alleviates neuronal apoptosis, and improves locomotor functional recovery by activating the AMPK/SIRT1 pathway and inhibiting NF-*κ*B activation in mice after SCI. Our results support the antineuroinflammatory effect of sesamol in SCI, and sesamol may be a potential therapeutic agent for the treatment of SCI.

## Data Availability

All data supporting the conclusions of this manuscript are provided in the text and figures. Please contact the author for data requests.
